# A mixed-methods exploration of virtual reality as a tool to promote green exercise

**DOI:** 10.1038/s41598-022-09622-x

**Published:** 2022-04-05

**Authors:** G. Calogiuri, B. J. Keegan, S. L. Birkheim, T. L. Rydgren, O. E. Flaten, F. Fröhlich, S. Litleskare

**Affiliations:** 1 Center of Health and Technology, Department of Nursing and Health Sciences, University of South-Eastern Norway, Drammen, Norway; 2 Department of Public Health and Sport Sciences, Inland Norway University of Applied Sciences, Elverum, Norway; 3 School of Business, Maynooth University, Maynooth, Ireland; 4 Department of Health and Nursing Sciences, Inland Norway University of Applied Sciences, Elverum, Norway; 5 Department of Digitalisation and Infrastructure, Inland Norway University of Applied Sciences, Elverum, Norway; 6 Department of Game Development - The Game School, Inland Norway University of Applied Sciences, Hamar, Norway

**Keywords:** Human behaviour, Public health, Risk factors

## Abstract

The salutogenic effects of green exercise are widely recognised, yet many individuals do not engage in this health-related behaviour. Using a convergent mixed methods approach, this study explored the impact of experiencing nature through Virtual Reality (VR) on the decision-making process relating to green exercise. Three experimental trials were conducted (overall *n* = 136), in which healthy adults were exposed to different VR scenarios reproducing a virtual walk in an existing urban green area. Participants reported medium–high rating of intent to visit the location. Significant pre-to-post increments in future green exercise intention were observed after the VR exposure, though a significance difference was not achieved in comparison with a control condition. Qualitative analysis revealed the impact of the VR experience on behaviour regulation, and highlighted the pivotal role of anticipated emotional benefits. Despite scepticism, the VR experience was effective in arousing curiosity to explore natural environments, which was associated with environmental perceptions as well as nostalgic and socio-cultural perspectives.

## Introduction

### The salutogenic effects of nature and green exercise

Natural environments provide great value for the health and well-being of people, as spending time in contact with nature provides benefits to people’s physical and psychological health. For instance, literature reviews show evidence of positive associations of nature exposure or interactions with indicators of physical health, such as biomarkers of immune function^1^, obesity and obesity-related diseases^2^, and incidence of other chronic diseases^3^. Moreover, a recent study across 18 countries found that people who live in neighbourhoods with greater availability of natural environments (including both, “green” and “blue” settings) reported higher levels of well-being^4^. It has been suggested that, in order to maintain higher levels of general health and subjective wellbeing, people should spend at least 120 min a week in nature^5^. Green exercise (i.e., physical activity taking place in presence of nature^6^) is particularly beneficial in this regard, as it combines the synergic benefits of physical activity with those provided by nature exposure^7,8^.

Green exercise can provide health benefits above and beyond physical activity taking place indoors or in urban settings^9,10^. A 2016 study estimated that the health benefits of green exercise saved society around £2.2 billion in the UK alone through welfare gains^11^. Green exercise, in different forms, has been also shown to be an effective preventative intervention in vulnerable groups (see e.g.,^12,13^) as well as an effective supplement in the treatment of different clinical conditions (see e.g.,^14–16^). Promotion of green exercise is thus important to achieve public health goals^3^, and visits to local natural environments should be encouraged in the population. In this respect, while there is large consensus on the importance of interventions that act on the physical and perceived environment, such as increasing accessibility to and safety of local natural environments^3,17^, initiatives that focus on behaviour changes have been also proposed as effective strategies to promote green exercise^18–20^. In particular, as virtual reality (VR) technology is becoming increasingly popular and economically accessible, it may represent an additional tool to complement the current initiatives to promote green exercise.

### Virtual reality and its potential to promote green exercise

VR is defined as a computer simulation that senses the participant’s position and actions and replaces or augments the feedback to one or more senses, giving the feeling of being immersed or present in the virtual world)^21^. In recent years, the application of this technology to recreate virtual simulations of natural environment has generated increasing attention as a way to deliver nature experiences to individuals who, for medical reasons or other barriers, do not have the possibility to access actual natural environments, or to contribute to reconnecting people with the natural world^22–24^. Evidence from studies in the context of nature-based tourism indicate that VR-based advertising may be an effective tool in promoting visits to natural destinations and enhancing attitudes towards green spaces^25–27^. An element that may play an important role in the effectiveness of VR-mediated nature experiences as a tool to promote green exercise, is the concept of *presence*. Presence is a person’s “(psychological) sense of being in the virtual environment”^28^. While it is primarily a subjective experience, presence relates to several aspects of VR technology. Firstly, presence relies on the technology’s ability to replace sensory input from the real world with sensory feedback from the virtual environment, often referred to as immersion^29,30^. When this sensory feedback, in combination with the content of the environment, creates a believable environment it contributes to higher levels of *place illusion*^29,30^. *Plausibility illusion* occurs when the events in the virtual environment are perceived as if they really were happening^29^. Lastly, the user may navigate the environment as a virtual avatar, or being able to interact with the environment using virtual hands, to allow *embodiment illusion* to occur (also known as the illusion of body ownership)^29,30^. All of these different types of illusions are believed to increase the levels of presence. Studies that have investigated the potential of VR as a tool in nature-based tourism have indicated that higher levels of presence are crucial for information-processing, attitude formation, and affordance^25,27^. Yet, to the best of the authors’ knowledge, little evidence exists on the extent to, as well as the psychological processes through which VR may promote green exercise as a health-related behaviour.

### Possible pathways explaining behaviour-regulation processes associated with VR

In order to understand the behaviour change processes elicited by VR-mediated nature experiences, it is important to consider the function of emotions on human behaviours, alongside the anticipated emotional benefits triggered by experiencing nature through VR. According with Baumeister et al. (2007)’s Feedback Theory, emotions have the function to provide feedback to which behaviours one should or should not pursue in future. In this context, *anticipated* emotions are seen as more important in guiding behaviour than actual, experienced emotions^31^. Although in some conditions emotions may have a direct causation on behaviours (such as in “fight or fly” situations), in more sophisticated behaviours the decision-making process is rather conscious, involving the evaluation of past experienced emotions, as well as influences from a person’s cultural and social contexts. It is especially important that a person anticipate powerful emotional outcomes^31^, which, in the context of VR-mediated nature exposure, may be facilitated by high levels of presence (see previous section). While experiencing nature through VR may elicit anticipated emotional benefits on the base of generalized previous experiences of green exercise (i.e., enjoyment and relaxation), such anticipated psychological benefits could be also seen through the prism of *nostalgic reconsumption of places*. Place consumption refers to the act of a cultural event or experience that is carefully created by a ‘producer’ (e.g., tourism board) for the benefit of a ‘consumer’ (visitor) and these actors feed off each other in a circuitous fashion^32^. Moreover, reconsumption refers to the active and conscious decisions to seek an experience again^33^. A poignant theme in this field refers to the interactions with physical places and displays significant connotations for “revisiting a fondly remembered place”^34^. In essence, a visitor to a place will develop place attachments through their interaction with a range of stimuli^35^, that leads to the creation of place experiences which fuel nostalgic reconsumption of places^34^.

### The present study

In recent years, studies on the effectiveness of VR-mediated nature experiences in different clinical contexts have been proliferated^22^. Although conceptual analyses have proposed VR as an effective tool in behaviour-change interventions, specifically in the promotion of green exercise^24,36^, the scientific evidence in this field remains scarce. Some studies, mainly within the field of tourism, have demonstrated that viewing naturalistic locations through VR can elicit intent to visit those locations^25–27^. However, to the best of the authors’ knowledge, studies specifically investigating this phenomenon in a behaviour-change perspective are still missing. In order to address these gaps, as an explorative study, the present paper is set to shed light on this under-researched subject. The overarching purpose of the study was two-fold: (i) to explore the potential of VR-mediated nature experiences as a tool to promote green exercise, and (ii) appraise the pathways explaining behaviour-regulation processes associated with VR-mediated nature experiences, as well as the participants’ perception associated with different ways of developing and delivering such experiences.

The study is based on data retrieved from three experimental trials, during which healthy adults were exposed to a 10-min VR simulation reproducing a walk in an existing naturalising location (an urban green space)^37–39^. Across the three trials, different VR scenarios were developed in different ways –most were 360° videos filmed to provide a first-person perspective of a walk in the nature environment, while one was a computer-generated model developed using photogrammetry and three-dimensional (3D) modelling techniques to reproduce a digital twin of the location. Different 360° videos were filmed, which were characterized by different levels of scene-stability (“unstable” vs. “stabile”), depending on whether or not the 360° videos contained scene oscillations associated with the cameraman locomotion, corner turns, or involuntary vibrations. The 3D model was also designed to have high levels of scene-stability. The VR scenarios were administered, in different combinations, during either a “sedentary” or an “active” exposure (i.e., sitting on a chair vs. self-paced walking on a manually-driven treadmill, respectively). Details regarding the trials, experimental conditions, and VR technologies employed are presented in Tables 1 and 2. Using a convergent mixed-methods approach, quantitative measurements of the participants’ intention to visit the location and possible changes in future green exercise intention were collected, as well as qualitative information on their perceptions and experiences relative to the VR experience.

**Table 1:** Overview of experimental conditions in the different studies.

Study	Design	Condition’s name (*n*)	Description	Duration	Activity level (*Mnd* [*IQR*])	Cybersickness (*Mnd* [*IQR*])
Study 1^38^ *Data collected in May 2017*	RCT with cross-over design and counterbalanced administration of conditions (15- to 30-min wash-out period). Participants visited the location in reality prior the experimentation	Sedentary*unstable 360° video (*n* = 26)^a^	VR exposure while sitting on a chair	10 min	Sedentary (RPE = 8.00 [6.00–11.00])	Severe (11pt scale = 8.50 [3.75–10.00])
		Active*unstable video (*n* = 26)^a^	VR exposure while walking on a manually-driven treadmill	10 min	Light-intensity (RPE = 11.00 [9.00–13.00])	Severe (11pt scale = 7.50 [6.00–9.00])
Study 2^39^ *Data collected in June-October 2018*	Single blind RCT with parallel groups (randomization based on pre-established order)	Sedentary*unstable 360° video (*n* = 25)	VR containing oscillations on the horizontal and vertical axis (e.g., due to cameramen locomotion and unwanted vibrations)	10 min	Sedentary	Severe (SSQ = 33.66 [14.96–99.11])
		Sedentary*stable 360° video (*n* = 25)	VR containing almost no oscillations by using a dolly and an electronic gimbal while recording	10 min	Sedentary	Concerning (SSQ = 18.70 [1.87–35.53])
Study 3^40^ *Data collected in August-December 2020*	Single blind RCT with parallel groups (“pick from a hat” randomization). Participants were exposed to stress-elicitation prior the condition by viewing a 2′50’’ film clip (Ray & Gross, 2007)	Active*stable 360° video (*n* = 20)	VR developed as a 360° video whilst walking on a manually-driven treadmill	10 min	Light-intensity (RPE = 9.50 [7.00–13.00])	Severe (SSQ = 24.31 [15.43–33.66])
		Active*stable 3D model (*n* = 19)	VR developed as a 3D model whilst walking on a manually-driven treadmill	10 min	Light-intensity (RPE = 12.00 [10.00–13.00])	Some. symptoms (SSQ = 14.96 [11.22–33.66])
		Control (*n* = 21)	Walk on a treadmill whilst staring a blank wall	10 min	Light-intensity (RPE = 11.00 [8.00–13.00])	Some symptoms (SSQ = 11.22 [3.74- 22.44])

RPE = Ratings of perceived exertion (6 = “Rest”, 9 = “Very light”, 13 = “Somewhat hard”). 11pt scale = Single item “I got dizzy during the virtual walk”, 11-point Likerst scale (0 = Not at all; 5 = Neutral; 10 = Absolutely). SSQ = Simulator-Sickness Questionnaire (10–15 = Some symptoms; 15–20 = Concerning, > 20 = Severe [Kennedy et al., 2003]). NB: the SQQ include “fatigue” and “sweating” as symptoms, which may have inflated the total score for the “Active” conditions as well as the “Control”.

^a^Participants are the same in both conditions as according to cross-over design.

**Table 2:** Overview of technology and techniques employed to develop and deliver the VR scenarios in the different studies.

Study	Type of VR	Development of the VR scenario	Soundscape	Playback	Interactivity
Study 1^38^	360° video	Samsung gear 360 sm-c200 camera mounted on a modified Yelangu s60t handheld stabilizer. Post-production editing done in Adobe After Effects CC 2017, Warp Stabilizer VFX, and in Samsung Gear 360 ActionDirector, build 1.0.0.2423	Audio recorded simultaneously by the camera’s microphone	Samsung S7, with Android 7.0, mounted on a Samsung Gear VR mask with Sennheiser HD 201 headset. In the “active” exposure, the participants walked on a manually-driven treadmill (Woodway, Curve) at a self-paced speed	﻿The participants could not influence the speed of the “virtual walk” No avatar
Study 2^39^	360° video	Unstable 360° video: Montage based on the 360° video developed for Study 1 Stable 360° video: Samsung gear 360 sm-c200 camera mounted on a Guru 360 camera stabilizer, with the cameraman standing on a dolly pushed by an assistant Both conditions: video montage and colour editing done in the VeeR Editor (VeeR, ZhongGuanCun, China) application directly on the Samsung S7	Audio recorded simultaneously by the camera’s microphone	Samsung S7, with Android 7.0, mounted on a Samsung Gear VR mask with Sennheiser HD 201 headset	The participants could not influence the speed of the “virtual walk” No avatar
Study 3^40^	360° video	GoPro Fusion (5228 × 2624, resolution, 30 frames per second; GoPro, San Mateo, California, USA), with the cameraman standing on an electric hoverboard (AAG, MADD gear electric, Victoria, USA). Post-production editing done in GoPro Fusion Studio (to apply the Full Stabilization filter) and Adobe Premier (to adjust over-saturated colours)	Audio recorded using a surround microphone with four channels (Zoom H2, Zoom Coorporation, Chiyoda-ku, Japan)	HTC Vive Pro HMD (field of view of 110˚; resolution of 2880 × 1600; refresh rate of 90 Hz) connected to a computer (Intel(R) i7-8700 k processor, 16 gigabytes of RAM, NVIDIA Geforce RTX 2080 graphics card), and Sony WH-1000X M3 noise-cancelling headphones (Sony Corporation, Tokyo, Japan). The VR system was connected with a manually-driven treadmill (Woodway, Curve) through a USB output, so that the participants’ pace was in sync with the movements in the virtual world	The participants could control the movements in the virtual world through the treadmill No avatar
	Computer-generated (3D model)	Terrain model obtained from hoydedata.no. Path and immediate surroundings scanned with a drone (Phantom 4 Pro UAV, DJI, Shenzhen, China) in 4 K resolution. 3D model reconstructed from the aerial photographs with the photogrammetry software RealityCapture (Capturing Reality, Bratislava, Slovakia)			

Demo videos of all VR scenarios are available online at https://www.youtube.com/channel/UCbzH1x--keKGwlnpwNRWUxA/videos.

## Results

### Quantitative findings

**Figure 1:**
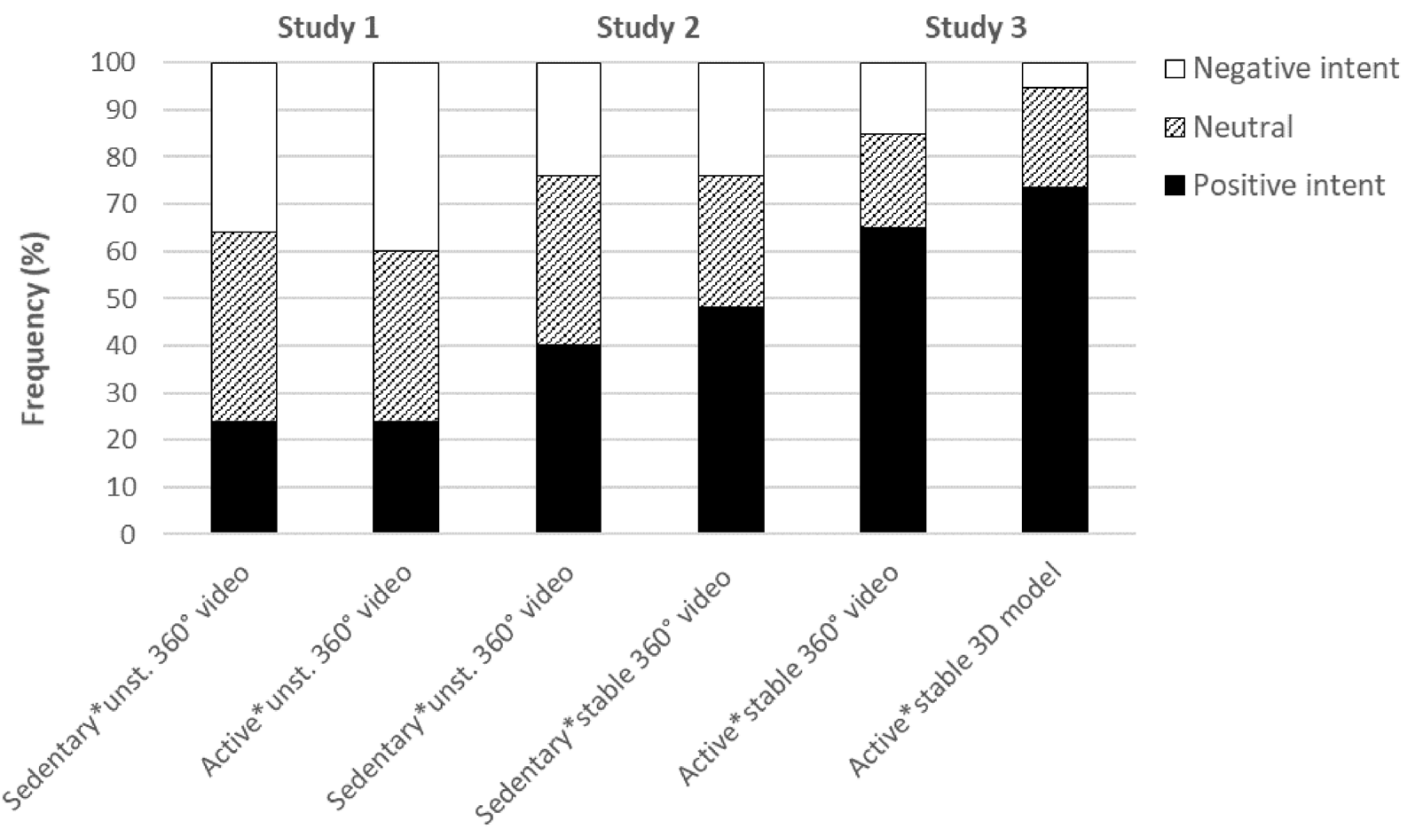
Intention to visit the location shown in the VR scenario (agreement with the statement “After the ‘virtual walk’, I now want to visit that place in the reality”). The intention to visit the location was rated on a 0–10 scale, but for the sake of simplicity the ratings were here conflates as “Positive intention” (rating 0 to 4), “Neutral” (raing point 5, which corresponds to the varbal cue “neither agree neither disagree”), and “Negative intention” (ratings 6 to 10).

Figure 1 shows the distribution of the participants’ ratings of intention to visit the real location reproduced in the VR scenarios across the different experimental conditions –each experimental condition representing a different combination of different ways to develop the VR scenario (“360° video” vs. “3D model”), levels of scene stability (“unstable” vs. “stable”), and mode of delivery (“sedentary” vs. “active”). In all the experimental conditions tested in Study 1 and 2, the ratings of intention to visit the real location were distributed around intermediate levels, indicating that less than half of the participants reported a positive intention to visit the location. More specifically, the median (*Mdn*) ratings were 5.00 (inter-quartile range [*IQR*] = 4.25) in both the “sedentary*unstable 360° video 1” and the “active*unstable 360° video”, while they were 5.00 (*IQR* = 4.00) and 5.00 (*IQR* = 3.00) respectively in the “sedentary*unstable 360° video 2” and “sedentary*stable 360° video”. The median ratings of intention to visit the real location ﻿were higher in both the experimental conditions tested in Study 3, which were the “active*stable 360° video” (*Mdn* = 7.00, *IQR* = 3.00) and the “active*3D model” (*Mdn* = 7.00, *IQR* = 4.00). In these conditions, overall, more than two thirds of the participants (*n* = 27) reported ratings above 5, indicating positive intention to visit the location, while only four participants reported ratings below 5 (neutral or negative intention). No significant differences were found between conditions within any of the individual studies. Significant differences were found between men and women in Study 2 (*U* = 190.00; *p* = 0.018), with higher values among the women (*Mdn*: 7.00, *IQR* = 5) compared to men (*Mdn*: 5.00, *IQR* = 2.00). Such sex-related differences were not replicated in Study 1 and 3. No significant correlations were found regarding the participant’s age or body-mass index (BMI) in any of the studies.

**Figure 2:**
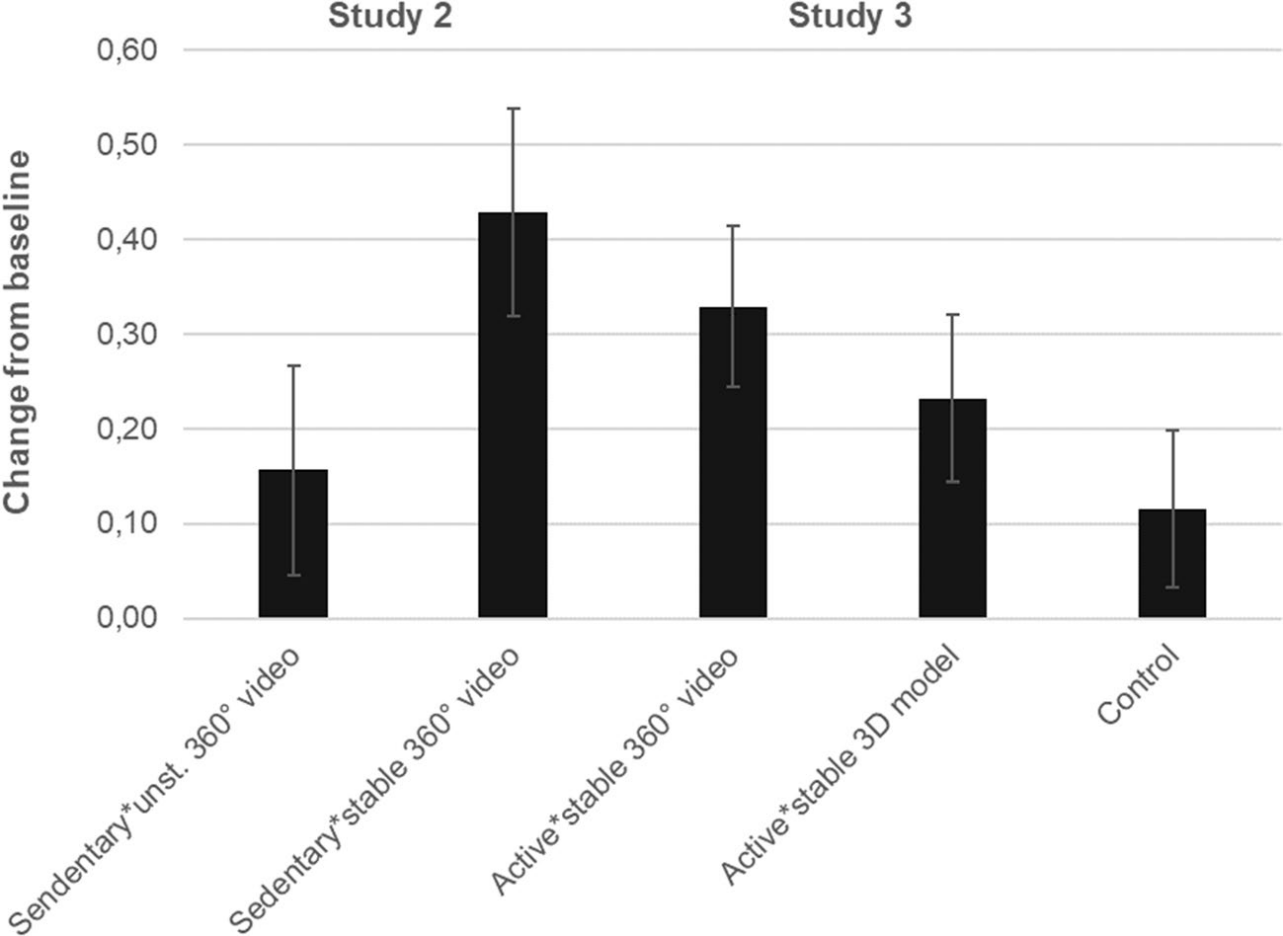
Changes from baseline in general green exercise intention, measured using the *Intention to Perform Green Exercise Questionnaire*^41^ before and after the VR exposure, in relation to different experimental conditions (EMM ± SE, as corrected for PA-levels). Left = Study 2; Right = Study 3.

Figure 2 shows the estimated marginal means (*EMM*) and standard errors (*SE*) of general green exercise intention expressed as change from baseline (delta values), corrected for the participants’ physical activity levels. In Study 2, the mixed between-within subjects analysis of co-variance (ANCOVA) on green exercise intention, corrected for physical activity levels, found a statistically significant within-subjects effect (*F*(1,47) = 5.570; *p* = 0.022; *Partial η*^*2*^ = 0.106) and a significant time by condition interaction (*F*(1,47) = 6.200; *p* = 0.016; *Partial η*^*2*^ = 0.117). The pairwise comparisons of the *EMM* demonstrated a significant increment of green exercise intention from baseline in the “sedentary*stable 360° video” condition (*p* < 0.001), but not in the “sedentary*unstable 360° video” condition (*p* = 0.981). In Study 3, the ANCOVA found a statistically significant within-subjects effect (*F*(1,56) = 7.016; *p* = 0.010; *Partial η*^*2*^ = 0.111), though no statistically significance was found for the time by condition interaction (*F*(2,56) = 0.126; *p* = 0.882; *Partial η*^*2*^ = 0.004). However, the pairwise comparisons of the *EMM* showed no significant changes from baseline in the control condition (*p* = 0.062), while a significant increment was found in the “active*stable 360° video” condition (*p* = 0.014) alongside a borderline significant increment in the “active*3D model” condition (*p* = 0.054). When examining the delta-values for green exercise intention in relation to the participants’ background information, no significant differences were found between men and women, nor were significant associations with the age and BMI found, in any of the studies.

### Qualitative findings

Three themes emerged through the analysis of qualitative findings. Firstly, participants presented diametrically opposed views towards VR as a tool for the promotion of green exercise. Secondly, the anticipated emotional outcome of the experience featured heavily in participants reflections. Thirdly, a rich vein of interpretations were provided in light of eliciting curiosity for exploration or nostalgic reconsumption of green spaces.

Mixed views surfaced regarding VR as tool for the promotion of green exercise. Participants displayed positive affirmation of their intention to engage in green exercise and exploration of green space:

“It is a great idea! With more research and better technology, I think that it can be an amazing tool to motivate people to exercise.” (Male, 23)

Critical reflections towards VR-mediated nature experiences were also evident, in particular related to the extent of the performativity of the VR experience. In one poignant example, a participant was unimpressed with the experience, suggesting that nothing compares to the experiential consumption of real nature:

“Walking outdoors should be an experience, and if you get that feeling by sitting inside on the sofa, you have made a fool of yourself.” (Male, 21)

Aside from the negative connotations expressed, analysis revealed how the locus of the argument stems from an intense appreciation of nature. Many comments such as this espoused a consistent perception of VR through a sceptical lens based on premise that it does not compare to the real life experience. This poses an intriguing conundrum for the role of VR as a promotional tool for visiting natural environments, as participants viewed it as simply a replacement for the inability to visit such a place. Similarly critical commentary on physical experiential aspects of the VR tool was commonplace. A focus on the audio-visual and sensory aspects were evident, and the resulting effects these had on the participants, such as dizziness and nausea.

“It will not be especially motivating if you get this feeling of nausea. That will not give you the motivation to continue”. (Female, 39)

Again this reflection is grounded on a highly personal motivation for experiential consumption of natural environments. This suggests that, while the audio-visual and sensory aspects of the VR have an impact on the enjoyment of the experience, they did not affect the participants’ overall attitudes toward its use as a persuasive strategy. Hence critical comments such as these offer promising results in terms of the viability of VR as a promotional tool for green exercise.

On the other hand, participants indicated a range of sensory elements which were key to their enjoyment of the VR experience. Most notably, the presence of other people in the VR scenario was a motivating factor for influencing participants’ intentions:

“When I see others in the video, I want to go outdoors with them and feel the nature”. (Male, 23)

Whereas exposure to the VR scenario from a sedentary position was seen as not truly reflective of active experiences in nature.

“The sitting condition was the worst because what happened on the screen did not match with what I did. The treadmill VR will probably motivate people to go more out in the nature.” (Male, 25)

In this respect, the VR exposure was shown to elicit a desire for green exercise through novel and fascinating environments in order to trigger the curiosity to visit the space that was virtually created.

The second theme which emerged is the impact of anticipated affective benefits resulting from the VR experience. Crucially, participant’s insights offered an understanding of the mechanisms of experiencing nature through VR as a tool for green exercise promotion:

“It contributed to my desire to walk along that road on one of my walks later.” (Male, 21)

Remarkably, such salutogenic benefits were reported by participants who presented positive affirmation as well as sceptical attitudes towards VR technology, underlining the potential for VR as a promotional tool for green exercise.

“Now I feel like going for a walk in real nature because I thought it was comfortable and it had a positive effect on my mood.” (Male, 23)

In light of the desired outcomes for exposure to the VR experience, it is clear that the tool is effective in arousing desire for green exercise, satisfying motivations for salutogenic reasons.

Thirdly, participants made explicit reference to the fact that the VR experience was effective in eliciting curiosity for exploration of naturalistic locations, including places which were previously visited.

“If you get to know a new place using VR, someone might get curious to visit the place in reality.” (Male, 27)

“The virtual walk made me want to visit the area that was reproduced in VR in real life. I also want to spend more time in nature in my own local area when I get the opportunity to do so.” (Female, 24)

With these findings, there are clear connotations for reconsumption of places. In that the desire to revisit a place or experience, in this case a natural environment near the testing site, was a strongly held view as a result of the VR exposure. Reconsumption practices were also evident through participant descriptions of the testing site, suggesting that the real nature exposure was a dominant factor in the mind-set of the users. In this manner, analysis of findings revealed how a significant proportion of participant motivations to visit and revisit the location were derived from personal interpretive reasons such as nostalgia. The role of nostalgia is clear in many participant reflections, whereby previous memories from visits to places similar to the virtual environment featured in their reflection on the experience.

“Most of all, I feel like I miss summer / spring, when I go for a walk every day.” (Male, 29)

“I did not feel that I came into more contact with nature, but maybe I would have done so if the environment was, for example, forest or mountains.” (Male, 22)

On the other hand, participants exhibited highly personal reflections on natural environments, which extended beyond the realm of nostalgia.

“What is a little fascinating is that it feels a bit like walking there for real, but the graininess of the film ruined it. There could have been slightly louder sounds from the wind, which would have made the experience more real.” (Female, 63)

“It was interesting to see the wind blowing through the trees without any other senses being able to help determine how much it was blowing.” (Male, 25)

In this respect we see an intriguing juxtaposition whereby participants are motivated to the point of constructing a personal version of the naturalistic location represented in the VR scenario. Considering how the virtual environment was constructed by the research team to reflect the natural space as closely as possible, it highlights the role of personal introspection in the construction of realities. This is a warning call for future use of VR, in that the most scientifically accurate replication of a natural environment may not match up to the highly subjective personal interpretation of the ‘perfect’ natural environment.

## Discussion

### Effectiveness of VR-mediated nature experiences in promoting green exercise

The quantitative findings of the present study provide indications that VR-mediate nature experiences can be, though to a limited extent, an effective tool to promote green exercise, eliciting intention to visit natural locations as well as enhanced general intention to engage in green exercise. While this corroborates and extends previous studies in the context of nature-based tourism^25–27^, on the other hand it is important to notice that there was a large variation in the participants’ intention ratings. Moreover, although the increases in intention appeared to be more pronounced after the VR conditions compared with a non-VR control, a statistical significant difference was not achieved between these conditions. Partially confirming these quantitative findings, the qualitative analysis revealed participants had diametrically opposed views on whether VR could be an effective tool for promotion of green exercise. At the same time, the qualitative findings provided further expansion on the underlying motivational processes that may link VR-mediated nature experiences to green exercise promotion.

### Anticipated psychological benefits

The qualitative work performed revealed substantial appreciation of the salutogenic effects of green exercise despite mixed opinions in relation to the quality of the VR experience. These findings expand on the quantitative findings, providing an insight into the underlying mechanisms that link VR experiences to green exercise intentions. Moreover, this displays a direct contribution to Feedback Theory, showing how anticipated emotions from physiological and cultural dimensions were elicited through the VR experience^31^. Notably, we highlight a significant degree of scepticism towards VR experiences, whereas this serves as a reminder of user’s appreciation and reminder of the salutogenic effects of visiting nature. This supports the assumption that experiencing nature through VR can produces powerful anticipated emotional outcomes in the users, as well as offer a contentious ‘talking point’ that affirmed participant views on green exercise. In this respect, an interesting perspective may be provided by viewing such trigger for appreciation of nature under the prism of the *perceived environmental restorativeness* (i.e. the extent to which an environment is perceived by a viewer as possessing characteristics that can elicit psychological restoration^42^. In previous studies, it was shown how perceived restorativeness is replicable in a virtual green exercise setting^37^, thought this was not associated with *actual* psychological benefits, such as enhanced positive affect of reduced fatigue^37,38^. This addresses a gap within the understanding of perceived restorativeness and its value for eliciting (and, possibly, quantify) *anticipated* psychological benefits in the context of (virtual or actual) green exercise experiences, as opposite to traditional perspectives that focus on *actual* psychological benefits (see e.g.,^42–45^).

### Nostalgic reconsumption of places

Reconsumption studies have previously focused mainly on habitualistic rituals^33^ and interaction and exposure to physical experiences^32^, and there are no significant studies in relation to interpersonal motivations to visit (or revisit) a natural environment through VR-mediated stimulus. Not least, place consumption stimuli, physical or otherwise, is also yet to be understood. The qualitative findings provide insights into the underlying motivational processes that link VR exposure to green exercise promotion via nostalgic reconsumption of places. In doing so, the findings also contribute to the ongoing debate around place reconsumption. By unveiling the conscious and unconscious decisions to engage in green exercise, our findings contribute with prior reconsumption studies^33^. Moreover, by focussing our attention on participants desire to revisit a physical environment subsequent to exposure to VR, we extend the work of Cervellon and Brown (2018)’s interpretation of ‘*fondly remembered places*’^34^. Considering place reconsumption has ramifications for place branding^32^, our work extends our understanding of desires and motivations for visitors to green spaces within places. Hence, we have shown that VR can be powerful tool in eliciting desire to visit and re-visit a natural space.

### Technical aspects of the VR technology

Although a direct statistical comparison across the studies (and thus across all VR conditions) was not possible, from the qualitative and, to a limited extent, the quantitative findings it would appear that the characteristics of the VR substrate can play a role in the resulting experience and consequent behaviour-change. In particular, some indication emerged suggesting that “active” VR exposures (e.g., VR combined with treadmill walking) may be more effective than “sedentary” ones (i.e., viewing an VR whilst sitting on a chair). Not only was the benefits of “active” exposures explicitly mentioned by the participants in the qualitative interviews, but it would be supported by the quantitative findings indicating greater levels of intention in the active exposures of Study 3. As a possible mechanism to explain this, it could be argued that, by reproducing the physical act of walking, the active exploration may allow for higher levels of presence through increased *plausibility illusion.* Moreover, motion-coupled VR experience are known to ease the feelings of discomforts that are often experienced when the visual and input doesn’t match the motor-proprioceptive input (also known as sensory conflict)^46^. However, it is important to take into account that other factors may have acted as confounders. Firstly, although all the 360° videos were filmed at the same time of the year (May), because of climate and weather conditions, the scenario used in Study 3 was characterized by greater levels of greenery and lusher vegetation compared to the scenarios used in Study 1 and 2, in which the trees and bushes did not fully develop leaves and the grass still had a number of brown patches. Hence, as the virtual environment was more aesthetically appealing, this may have contributed to the more positive intention rating observed in Study 3 than the previous studies. Secondly, compared with the previous studies, in Study 3, new-generation technology was used as a substrate for the VR exposure, resulting in better video resolution. Finally, the levels of scene stability (i.e., whether the VR scenario contained high or low levels of oscillations) in the different studies are also likely to have influenced both the overall experience and the outcomes in term of behavioural intentions. This is corroborated by both, the qualitative reports as well as the fact that the intention ratings tended to be higher in the VR conditions that included a scenario with higher levels of scene-stability (i.e., a stable 360° video or the 3D model). This can be explained by the fact that presence of oscillations in the VR scenarios, as in unstable 360° videos, can elicit cybersickness^47^. Cybersickness, a malaise that can occur in users exposed to different digital visual medium, can dramatically impact the users’ psychological responses to the VR experience^36^. Previously published findings^37,38^ demonstrated that some of the VR conditions included in the present study, which contained a greater degree of scene oscillations (i.e., the “passive*unstable 360° video” in Study 1 and 2, as well as the “active*unstable 360° video” in Study 2), were quite prone to elicit cybersickness in the participants. On the other hand, the VR conditions where maximal stabilization was applied (i.e., the “sedentary*stable 360° video” in Study 2 and the “active*stable 360° video” and “active*3D model” in Study 3), were associated with lower incidence and severity of cybersickness^38^. Altogether, this suggests that the effectiveness of VR-mediated nature experiences as a tool to promote green exercise may be more reliant on the quality of the technological substrate than the way it is administered. However, as we envisage that VR technology will improve, so will its application in the developing field of digital placemaking^48^ and health promotion^38^.

### Strengths and limitations

Strengths of the present study are its novelty and experimental rigour of the individual studies (laboratory-based RCTs, two of which applied blind allocation of participant to conditions). The use of a mixed methods methodology can be also seen as a strength, as this allowed both to establish statistically significant effects of the VR treatment, as well as provide an in-depth understanding of the participants’ perceptions and experience. Moreover, validated and theoretically sound instrument were used in the quantitative part, while a rigorous and transparent analytical approach was used for the qualitative findings.

A number of weakness, however, need to be taken into consideration. Firstly, as this was an exploratory study, the extent of the evidence presented in this paper is limited. This study was based on the analysis of secondary data collected within previous studies, which may have resulted in some biases —a common limitation with secondary analysis^49^. Moreover, the quantitative datasets from the studies could not be compiled together, limiting the possibility to perform statistical comparisons across studies. The duration of the VR exposure in all studies was 10-min, a time-span chosen as it has been previously found to be a minimum bout for achieving health and wellbeing benefits in relation to both nature exposure^40^ and physical activity^50^, whiles at the same time prevent (or limit) the insurgence of cybersickness^51,52^. This time span may have been, however, too short to generate deep feelings of presence, although scientific evidence on the minimum VR exposure time needed for generating meaningful feelings of presence is scarce. Another limitation is the fact that green exercise intention was used as an indicator of behaviour-change, whereas the effect of VR exposure on actual green exercise behaviour yet is unknown. The natural environment represented in the VR scenarios was rather simple (in Study 1 and 2, also characterized by low levels of greenery). While this choice was in line with our purpose of exploring the potential of VR in promoting green exercise as a health-related behaviour, encouraging people to visit local natural environments in their everyday life, this may have limited the potential of our interventions to elicit enhanced intentions (as compared, for instance, with what we may have achieved by using a more awe-like scenario). Only one of the studies (Study 3) included a non-VR comparison condition, hence the present study provides only limited evidence on the effectiveness of VR-based interventions as compared with other types of green exercise promotion initiatives. Finally, as Norwegians are known to generally appreciate green exercise^53^, interpretation of the findings should account for the cultural context where the study was conducted.

## Conclusions

The findings of the present study provide promising evidence on the potential of VR-mediated nature experiences as a tool to promote green exercise, as well as novel and valuable insights into behaviour regulation associated with VR exposure, highlighting the pivotal role of anticipated emotional benefits and nostalgic reconsumption of places. This knowledge can inform green exercise promotion interventions. In this respect, although this study focused on non-clinical populations, the knowledge generated may be applied to different clinical population who would particularly benefit from green exercise, such as individuals with mental health challenges or those in need of increasing their physical activity levels. More research is needed in this field. In particular, it is recommended that future research include non VR-related interventions as a comparison/control, as well as follow-up assessments of green exercise behaviour.

## Methods

### Study designs and data

We employed a mixed methods design^54,55^. The quantitative and qualitative strands were conducted and analysed in parallel, hence the results presented separately, in line with a “separative” relational dimension. Merging was performed using an explanatory unidirectional approach, with the qualitative findings enhancing and deepening the quantitative findings. This was facilitated by the fact that the quantitative and qualitative data were collected in the same experimental context, among the same participants, with the qualitative questions being purposefully developed based on the same overarching themes of the quantitative instrument. Interpretation and reporting was eventually done narratively using a weaving approach, presenting both qualitative and quantitative findings together on a theme-by-theme basis.

The data were retrieved from a larger study including three controlled randomized trials conducted between 2017 and 2020^37–39^. The primary goal of these trials was to examine the participants’ psycho-physiological responses to different ways of developing and delivering VR experiences, though additional measurements were collected to examine the potential of the VR-intervention as a strategy to promote visits to real natural environments and green exercise, which will be presented in this paper. All studies were conducted at the Sport physiology laboratory of the Inland Norway University of Applied Sciences – Campus Elverum (Norway), and were designed by the same principal investigator (GC) and laboratory coordinator (SL). Details about the research design of all individual studies are available in the main publications^37–39^, and summarized in Table 1.

### Participants

Participants (overall n = 136) were recruited among students and employees at the Inland Norway University of Applied Sciences – Campus Elverum and among other citizens living in the vicinity. In Study 1, the inclusions criteria were: being between 20 and 45 years old, being able to walk for 10 min outdoors, and not being an elite athlete. For Studies 2 and 3, the inclusions criteria were: being 18 years or older, have normal or corrected-to-normal sight, and not having any diagnosis of balance impairments. Quantitative data were collected among all participants, of whom 68 were men (age range: 19–63 y.) and 68 were women (age range: 17–67 y.). Most (though not all) individuals showed normative BMI values (men: 20.00–37.30; women: 17.23–34.30) and reported a rather large range of weekly physical activity (the Metabolic Equivalent of Task [MET] assessed by Leisure-time exercise questionnaire^56^ was 18–119 and 15–128 METs for men and women, respectively). A subset of participants (n = 65) partook in the qualitative study. The characteristics of this sub-sample were similar to those of the overall sample with respect to age (men: 19–63 y.; women: 19–67 y.), BMI (men: 20.00–37.30; women: 18.20–34.30) and weekly physical activity levels (men: 18–119 METs; women: 15–91 METs). No relevant differences across the studies were noticed with respect to participants’ characteristics, with the only exception for a lower mean age in Study 1 compared with Study 2 and 3, which was due to the more restrictive inclusions criteria relative to age. All studies were performed according to the Declaration of Helsinki and approved by the Norwegian Centre for Research Data (Study 1 and Study 2, ref. n. 53,246 and 60,451, respectively) or the Regional Committees for Medical Research Ethics South East Norway (Study 3, ref. n. 134,663). All participants were informed about the purpose of the study and associated risks, and provided an informed written consent.

### Experimental conditions and VR technology

All VR scenarios provided a first-person view of a nature walk in the exact same location, an urban green area with a walking path by the river Glomma in Elverum (Norway), though different techniques were used to develop and administer the VR scenarios in the different studies. Most of the VR scenarios were 360° videos, filmed to provide a first-person perspective of a walk in the naturalistic location, while one VR scenario was a computer-generated model developed using photogrammetry and three-dimensional (3D) modelling techniques to reproduce a digital twin of the location (demo videos of all VR scenarios are available online at https://www.youtube.com/channel/UCbzH1x--keKGwlnpwNRWUxA/videos). The 360° videos for the different conditions were developed using techniques resulting in lower or higher levels of scene-stability (i.e., the extent to which the 360° videos contained scene oscillations associated with the cameraman locomotion, corner turns, or involuntary vibrations). The 360° videos characterized by lower levels of scene-stability (“unstable”) were filmed using a mechanical camera stabilizer with the cameraman walking on a paved path across the naturalistic location. In the other 360° videos, higher levels of scene-stability (“stable”) were achieved through various techniques, including the use of a trolley or electric hoverboard for filming, mounting the camera on an electronic gimbal or applying a stabilization filter in post-production, and adding smooth transition scenes by fading in and out at corner-turns. The 3D model was also designed to have high levels of scene-stability (i.e., it did not contain oscillations associated with locomotion or sudden turns). It should be noted that the 360° videos for Study 3 was filmed with a “new generation” 360° camera, which resulted in a higher quality video resolution. Moreover, while the 360° videos were all filmed in the same period of the year (May), depending on the specific climate and weather conditions for each year, the scenarios contained different levels of greenery. These VR scenarios were administered, in different combinations, either during a sedentary (i.e., sitting on a chair) or an active (i.e., self-paced walking on a manually-driven treadmill) session. In the active conditions, the participants walked on a manually-driven treadmill (Woodway, Curve), which allowed the participants to walk at a self-paced speed, operating the treadmill directly with the movement of their feet. In both VR conditions of Study 3 (a 360° video and the 3D model), the treadmill was connected to the VR system through an USB output to match the speed of the playback with the speed of the treadmill. This procedure also allowed participants full control of the navigation speed of the virtual world by simply walking faster or slower on the manually driven treadmill. The self-pacing was preferred to a standardized speed not only for safety reasons (to avoid participants falling or stumbling, as the their eyes were covered by the VR headset), but also to provide greater autonomy during the VR exposure, an important factor supporting future physical activity behaviour^57,58^. The resulting combinations characterizing the different experimental conditions were the following:

- Sedentary*unstable 360° video (Study 1)
- Active*unstable 360° video (Study 1)
- Sedentary*unstable 360° video (Study 2)
- Sedentary*stable 360° video (Study 2)
- Active*stable 360° video (Study 3)
- Active*stable 3D model (Study 3)

Additionally, in Study 3, a control condition was also included, in which the participants walked for 10 min on the manually-driven treadmill without being exposed to VR. Each exposure lasted around 10 min to balance the benefits of nature exposure and exercise with the risk if inducing cybersickness. Previous research has found significant effects on exercise intention after as little as 5-min’ walk in a natural environment^59^, while reviews highlight that a duration of 10 min may be required to attain the benefits of both nature exposure^40^ and exercise^50^. Exposure beyond these minimum requirements were avoided due to the increased risk of cybersickness with prolonged VR exposure^51,52^. Within each study, no between-conditions differences were found with respect to ratings of perceived environmental restorativeness^37,38^. Information about the technology and techniques employed to develop and deliver the VR scenarios is presented in Table 2. Further details are available in the primary publications^37–39^.

### Quantitative measurements

The participants’ intention to visit the real location viewed in the VR scenario was assessed in all studies through a single item, which asked the participants to rate their level of agreement with the following statement: “After the ‘virtual walk’, I now want to visit that place in reality.” The participants reported their response on a 0–10 visual scale with anchor at 0 (= “Absolutely disagree”), 5 (= “Neither agree, nor disagree”), and 10 (= “Absolutely agree”). Although it has been recommended that scales for general intentions relative to people’s health-related behaviours are developed as a three-items scale^60^, single-items scales have been previously used to assess intention to exercise in specific locations (see e.g.,^59^).

The effects of being exposed to the VR condition on the participants’ general green exercise intention was assessed by administering the *Intention to Perform Green Exercise Questionnaire*^41^ before (pre) and after (post) undergoing each experimental condition. Green exercise intention was assessed only in Study 2 and 3, as the instrument was yet not available when Study 1 was conducted. The instrument is compounded of five items (e.g., “I intend to do green exercise”), each being rated on a 1–7 scale with anchor at 1 (= “Absolutely disagree”) and 7 (= “Absolutely agree”). The caption provided a specific time reference (“in the course of the next week”) and a definition of green exercise (“Examples of what is intended as green exercise: walking or exercising in parks, green- or natural environments. This can include commuting to and from work/school or walk/exercise with a dog or other domestic animal”). The instrument showed a high internal consistency in both studies and in both the pre- and post-exposure assessments (alpha = 0.94–0.95).

As past exercise behaviour influences future exercise intention and behaviour^61^, the participants’ physical activity levels were assessed at baseline (i.e., before the beginning of the experimentations), to be used as a covariate for green exercise intention. Physical activity levels were assessed using the Godin's Leisure Time Exercise Questionnaire^56^. The instrument assesses physical activity of different intensities (light, moderate, and vigorous) to generate an overall score in metabolic equivalent of task (MET). For the purposes of the present study, the caption was modified to include non-leisure forms of physical activity, such as active commuting –such modified version was previously found to be highly correlated with objective assessments of physical activity^62^.

### Qualitative information

Participant perspectives were collected through qualitative data via the survey instrument. The open-ended nature of the questions allowed participants to offer a personal reflection on the extent to which VR-mediated nature experiences encourages experiential consumption of green exercise. Questions were framed in line with the tenets of Feedback Theory regarding the emotional benefits of the VR experience. Additionally, participants were offered the opportunity to indicate the reasons why (or why not) they perceived the virtual walk as enjoyable. Questions were objectively phrased to allow for negative, positive as well as neutral viewpoints.

### Data analyses

#### Statistical analysis

The quantitative data were preliminary examined for frequency distribution, possible outliers, and missing values. Due to the differences in study design, experimental condition, and time of assessment, the data from the three studies were analysed separately. The participants’ ratings of intention to visit the location were examined using descriptive statistics and, as the data were not normally distributed, presented through Median (*Mdn*) with Inter-Quartile Ranges (*IQR*). According with the observed non-normal data distribution, Wilcoxon Signed Ranks Test (for Study 1) and Mann–Whitney test (for Study 2 and 3) were used to establish possible differences among the different experimental conditions within the individual studies. For the analysis of green exercise intention, which included pre and post assessments as well as between-groups comparisons, a mixed between-within subjects analysis of covariance correcting for the individuals’ physical activity levels (ANCOVA) was preferred in order to reduce the risk of Type I error. An exponential transformation was preliminary applied to the green exercise intention data in order to enhance the data’s negative skewedness. “Time” (pre/post assessment) was the within-subjects factor and “condition” (Study 2 = Sedentary*unstable 360° video, Sedentary*stable 360° video; Study 3 = Active*stable 360° video, Active*3D model, Control) was the between-subjects factor, while physical activity levels was set as a covariate. If statistical significance was achieved in the ANCOVA, a pairwise comparison of the estimated marginal means (*EMM*) of green exercise intention corrected for physical activity levels was performed to test for possible pre-post differences in the individual experimental conditions. A Bonferroni adjustment of alpha was applied to reduce the risk of Type I error due to multiple comparisons. Additional analyses were conducted in order to establish possible associations of intention to visit the locations or green exercise intention (the latter expressed as delta-values) with the participants’ background information (sex, age, and BMI). More specifically, Mann–Whitney test were used to establish possible differences between men and women, while Spearman’s Rank Correlation Coefficient was used to establish possible associations with the participants’ age and BMI. All statistical analyses were performed using IBM Statistics SPSS version 26 (IBM Corp., New York). Significance was set at *p* < 0.05.

#### Analysis of the qualitative data

**Figure 3:**
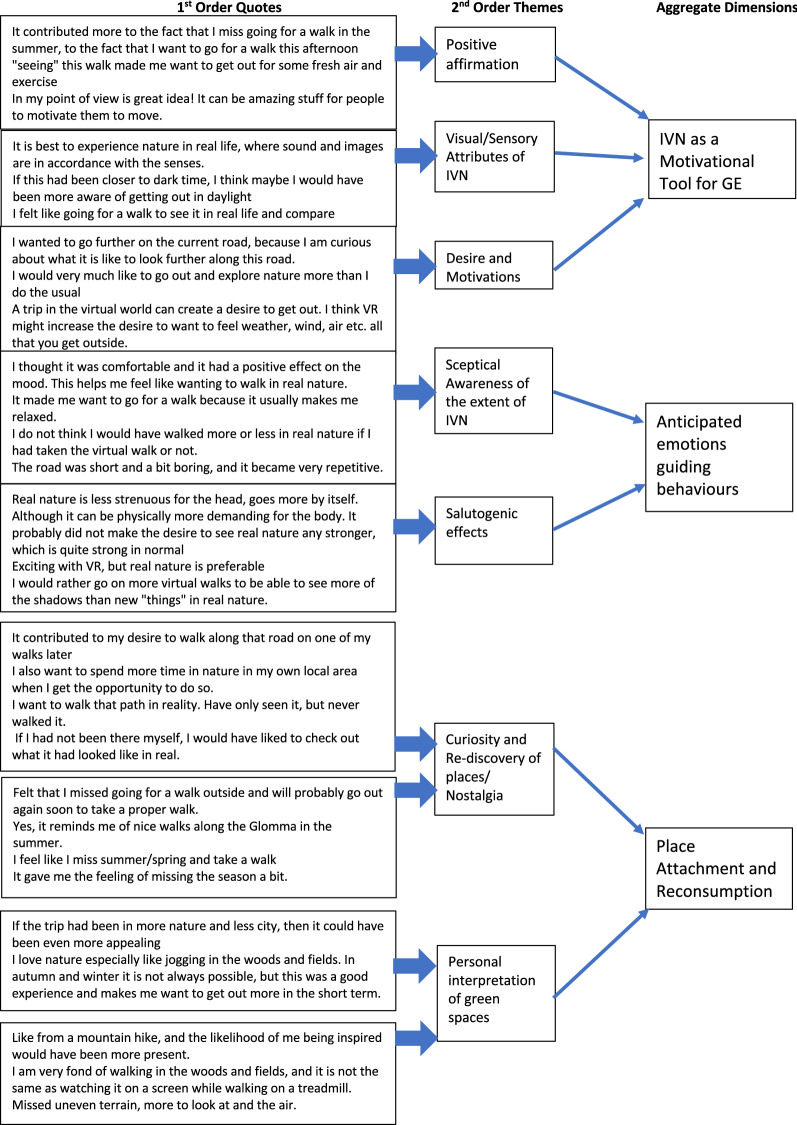
Example of second order themes and aggregate dimensions emerging from qualitative data.

The qualitative information was analysed by using the Gioia method^63^. Participants were invited to articulate their reflections and intentions which were gathered and analysed thematically into 1st and 2nd order constructs, leading to aggregate dimensions of the qualitative findings^63^. Following initial classification of the participant’s reflections, two researchers (B.J.K. and S.L.B.) independently coded the responses, which were reviewed by two further researchers (G.C. and S.L.), who sampled the combined coding to check consistency and pattern matching to ensure validity, as well as inter-coder reliability. Subsequently, the output of the coding process was interrogated to classify emergent patterns and themes. Following this, the researchers engaged in a series of hermunetical cycles of analysis noting repetitions in the data and corollary themes. The analysis operation led to the development of second order themes, and aggregate dimensions which are presented in Fig. 3.

### Ethics approval

All studies were registered at and approved by the Norwegian Centre for Research Data (Study 1 and Study 2, ref. n. 53,246 and 60,451, respectively) or the Regional Committees for Medical Research Ethics South East Norway (Study 3, ref. n. 134,663). All participants were informed about the purpose of the study and associated risks before they provided their written consent.

## Data Availability

The datasets of this study is not publicly available at the moment. All relevant aggregated data are provided in the paper. The data used in the current study may be made available after agreement with the authors.
